# Disease knowledge and health literacy in parents of children with sickle cell disease

**DOI:** 10.1002/jha2.762

**Published:** 2023-08-11

**Authors:** Bonaventure G. Ikediashi, Cristina Ehrmann, Selma Gomez, Gisela Michel

**Affiliations:** ^1^ Faculty of Health Sciences and Medicine, University of Lucerne Lucerne Switzerland; ^2^ Swiss Paraplegic Research Notwill Switzerland; ^3^ National Sickle Cell Disease Centre of Benin (New‐Born Screening of Sickle Cell Disease and Comprehensive Clinical Care Programs, CPMI‐NFED) Cotonou Benin

**Keywords:** Benin, disease knowledge, health literacy, hospital admissions, sickle cell disease

## Abstract

We aimed to (1) describe sickle cell disease (SCD) knowledge and health literacy levels in parents of children with SCD, (2) examine associations with socio‐demographic factors and (3) analyse the association with hospital admissions and frequency of occurrence of painful episodes. Parents who presented with their child at routine hospital consultation at the National Sickle Cell Disease Centre in Benin were administered a questionnaire assessing SCD knowledge, health literacy (newest vital sign [NVS]) and socio‐demographic and clinical characteristics. In total, 117 parents participated (108, 92.3% females). The predominant SCD genotype was HbSS (79.5%). The average SCD knowledge score was 13.6 (standard deviation [SD] = 2.0). Only 34 (29.1%) participants correctly answered ≥70% of the questions, indicating good knowledge. Health literacy was relatively low (mean NVS score = 3.3; SD = 1.1). SCD knowledge was higher in parents with older children (*p* = 0.001) and higher education levels (primary, *p* = 0.010; tertiary, *p* = 0.036 compared to participants with no formal education). Hospital admissions were more frequent when parents had lower SCD knowledge (*p* = 0.034) and in parents with younger children (*p* = 0.039). No associations were found between health literacy and hospital admissions (*p* = 0.940) and frequency of occurrence of painful episodes (*p* = 0.224). Continuous disease‐specific education for parents of children with SCD may help them better identify and prevent the occurrence of symptoms and decrease the number of hospital admissions.

## Graphical abstract

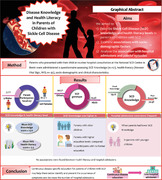

## 1 INTRODUCTION

Parents of children with sickle cell disease (SCD) are tasked with managing the condition of their children from infancy until adolescence. SCD is a genetic blood disorder that disproportionately affects people of sub‐Saharan Africa and those of African descent [1]. Globally, it is estimated that annually about 300,000 children are born with SCD, of which nearly 75% are found in sub‐Saharan Africa [2]. A recent study conducted in five sub‐Saharan African countries reported a mortality rate of up to 36% in children with SCD aged under 5 years [3]. In Benin, the prevalence of SCD is estimated at 4%, thus presenting an important healthcare burden in the country [4].

Patients with SCD can experience various medical conditions related to the disease [5]. Vaso‐occlusive crisis, which is a common phenomenon, is marked by episodes of excruciating pain and severe anaemia, therefore necessitating frequent hospital visits and admissions [6].

Parental health literacy has important implications for paediatric patients in general [7]. Adequate parental health literacy and disease knowledge are necessary for making informed decisions and taking appropriate actions that are beneficial to the child's health. Health literacy and disease knowledge are related but distinct concepts. Health literacy involves a range of skills and abilities that allow individuals to access, understand, evaluate and apply health information to make informed health decisions [8]. Disease knowledge specifically refers to the knowledge and understanding of a particular disease. Health literacy is a broader concept that encompasses disease knowledge as well as other skills. Assessing both concepts separately provides a more comprehensive understanding of the individual's skills and knowledge and allows for developing targeted interventions. In the context of a chronic condition such as SCD, this can include adopting preventive measures and early recognitions of signs and symptoms. SCD knowledge is believed to have an important influence on healthcare utilisation or hospital admissions [9]. Empowering parents of affected children with adequate knowledge, could ensure that they are better equipped to manage their children's condition [10].

Given the importance of health literacy and disease knowledge for health outcomes in chronic diseases, it is important to examine their relationship within an SCD population in the context of a low resource setting such as Benin in West Africa. Socio‐demographic factors such as age, gender, education and income level often reflect cultural and contextual factors that inform health beliefs, access to health care services and ability to use health information effectively [11, 12]. By considering these factors, we can better understand how cultural and contextual factors influence health literacy, disease knowledge and ultimately health outcomes in a population with a chronic disease [13, 14], such as SCD.

Few studies have assessed health literacy, disease knowledge and its impact on health outcomes in SCD, and most of them have been carried out in other contexts [10, 15, 16]. Studies carried out in sub‐Saharan contexts often focussed on perceptions and attitudes in a general population [15, 16, 17] but did not examine the impact of disease knowledge on health outcomes in SCD‐affected populations. To our knowledge, no studies have evaluated parental health literacy and disease knowledge in SCD in Benin, including its association with health outcomes and socio‐demographic factors.

Therefore, we aimed to (1) describe SCD knowledge and health literacy levels in parents of children with SCD, (2) examine associations between genotype, socio‐demographic factors, health literacy and SCD knowledge, and (3) examine the associations between parents’ SCD knowledge, health literacy, socio‐demographic factors and (a) frequency of hospitalisations and (b) frequency of occurrence of painful episodes. We hypothesised that the number of hospital admissions and frequency of occurrence of painful episodes are associated with parents’ SCD knowledge and health literacy levels. In addition, we hypothesised that SCD knowledge and health literacy levels are associated with socio‐demographic factors.

## 2 STUDY DESIGN AND METHODS

This cross‐sectional study among parents of children with SCD was approved by the National Ethics Committee in Health Research in Benin (CNERS Avis No. 43).

### 2.1 Study participants

To be eligible for this study, participants had to meet the following criteria: (i) have a child diagnosed with SCD and (ii) French language proficiency (to complete the health literacy questionnaire); however, this was not required for the disease knowledge questionnaire. Parents of children older than 13 years were excluded from participating in the study in order to focus specifically on the age group where parents and parental health literacy and disease knowledge play an important role in managing their child's health. A total of 117 participants were enrolled in this study between January and December 2021. However, 22 participants did not complete the health literacy questionnaire because either they did not have the required French language proficiency necessary to complete the questionnaire (*n* = 5) or they indicated that they could not complete the questionnaire (*n* = 17).

### 2.2 Procedure

Participants were recruited at the National Sickle Cell Disease Centre of Benin (New‐Born Screening of Sickle Cell Disease and Comprehensive Clinical Care Programs, CPMI‐NFED) using a convenient sampling method. CPMI‐NFED is the reference centre for the management and treatment of children and pregnant women with SCD in Benin. It is located at the University Teaching Hospital in Benin. The centre manages about 3000 patients. Patients aged 0–2 years are usually followed monthly, and those older than 2 years of age attend regular follow‐up visits every 3 months. Parents are educated about their child's disease when they enter follow‐up care. A trained research assistant approached eligible parents in the patients’ waiting area, while they were waiting to attend their routine consultations. After consenting to participate in the interview, by signing the consent forms, the research assistant asked participants the interview questions and documented the answers.

### 2.3 Measures

#### 2.3.1 Outcome measures

Two outcome measures of interest were collected: the number of hospital admissions (defined by hospital stays that included at least an overnight stay) and the frequency of occurrence of painful episodes (defined by pain that required medication or emergency hospital visits) in the last year. Both outcome measures were self‐reported by parents.

#### 2.3.2 Predictors

##### SCD knowledge

A locally developed disease knowledge questionnaire was used. It was developed in collaboration with SCD specialists and included topics they considered relevant for parents of children with SCD. The SCD knowledge questionnaire consists of 13 questions in total, with each correct answer assigned 1 point. Of these 13 questions, one had four answer options, while another had six answer options with each correct option answered assigned 1 point. Therefore, the participants could reach a possible maximum score of 21 points in the disease knowledge questionnaire. We considered 70% of correct answers to be good SCD knowledge. For participants who were uncomfortable with reading and writing in French, a research assistant helped to explain the questions in the local language.

##### Health literacy

The newest vital sign (NVS) is a rapid assessment tool appropriate for use in healthcare settings [18]. It is a frequently used tool for measuring health literacy in adult populations, and has been validated in several languages [18, 19, 20, 21]. The NVS questionnaire consists of six questions based on the labelling of an ice‐cream container. Each correct answer is assigned 1 point, and the health literacy score is calculated by summing up points based on correct answers. A person with a score of 0–1 is highly likely to have limited literacy, a score of 2–3 suggests the potential for limited literacy and a score of 4–6 suggests adequate health literacy. The French version of the NVS [22], was used for this study to evaluate the health literacy levels of the parents of the children with SCD because French is the official language in Benin. Participants who could not read and write in French were exempted from completing the questionnaire.

##### Socio‐demographic factors and clinical characteristics

Socio‐demographic characteristics included the age of the parent, education level attained (primary, secondary, or tertiary), employment status (salaried employment, self‐employed, not employed), and age and gender of the child. Clinical characteristics, that is, information on the genotype of the disease (HbSS, HbSC or others), were collected from the parents.

### 2.4 Data analysis

We used descriptive statistics to describe the overall level of health literacy and SCD knowledge, and socio‐demographic characteristics. To analyse the association between SCD knowledge and socio‐demographic characteristics, the following analysis steps were performed: (1) univariable regressions were carried out with SCD knowledge score as dependent variable and the health literacy score and socio‐demographic characteristics as independent variables. (2) Multivariable regressions were then carried out to examine associations between SCD knowledge and health literacy scores and socio‐demographic characteristics that were significant at *p* < 0.05 in the univariable analysis. This additional analysis was performed to help us identify possible confounders in the association.

Associations between outcome measures (hospital admissions and frequency of occurrence of painful episodes as dependent variables) and SCD knowledge, health literacy scores and socio‐demographic characteristics as independent variables were analysed using Poisson regression models. In the first step, univariable regressions were used, followed by multivariable regressions including measures significant at *p* < 0.05 level in the univariable regression.

## 3 RESULTS

### 3.1 Participants and their characteristics

Participant characteristics, health literacy scores and SCD knowledge are described in Table 1. In total, 117 parents participated. They were mostly females (92.3%), and nearly half of the children were female (*n* = 57, 48.7%). The overall mean age was 34.8 years (standard deviation [SD] = 5.9) for the parents and 8.7 (SD = 3.0) years for the children. The predominant genotype of the children was HbSS (79.5%). The average SCD knowledge score was 13.6 (SD = 2.0) and only 34 (29.1%) of the participants correctly answered at least 70% of the questions indicating good knowledge. The average health literacy score of the participants was 3.3 (SD = 1.1). Based on the health literacy (NVS) scoring, over half of participants scored between 2 and 3 points indicating a potential for limited literacy (*n* = 57, 60.0%). Most of the participants either reported having no (*n* = 53, 45.3%) or one hospital admission (*n* = 50, 42.7%). Most of the participants reported one (*n* = 50, 42.7%) or two painful episodes (*n* = 38, 32.5%) in the past 12 months.

**TABLE 1:** Descriptive demographic characteristics, genotype, disease knowledge scores and health literacy scores.

	*N* or mean	% or SD
Total number of participants	117	100.0
Parents’ gender (female) (*n*, %)	108	92.3
Child's gender (female) (*n*, %)	57	48.7
Parent's age (mean, SD)	34.8	5.9
Child's age (mean, SD)	8.7	3.0
Child's genotype (*n*, %)		
HbSS	93	79.5
HbSC/HbS beta thalassemia^a^	24	20.5
Parents’ education level (*n*, %)		
None	6	5.1
Primary	32	27.4
Secondary	62	53.0
University	17	14.5
Parents’ employment status (*n*, %)		
Salaried employment	26	22.2
Self‐employed	67	57.3
Unemployed	24	20.5
Parents’ health literacy scores (NVS) (mean, SD)	3.3	1.1
0–1 (highly likely to have limited literacy	2	2.1
2–3 (potential for limited literacy)	57	60.0
4–6 (adequate health literacy)	36	37.9
Parent's SCD knowledge scores (mean, SD)	13.6	2.0
≥70% correct responses (n,%)	34	29.1
Child's hospital admissions (past year) (*n*, %)		
0	53	45.3
1	50	42.7
2	10	8.6
3	4	3.4
Child's painful episodes (past year) (*n*, %)		
1	50	42.7
2	38	32.5
3	26	22.2
4	1	0.9
5	2	1.7

Abbreviations: NVS, newest vital sign; SCD, sickle cell disease; SD, standard deviation.

^a^ One patient with HbS beta‐thalassemia+ genotype.

### 3.2 Association between SCD knowledge, health literacy and socio‐demographic factors

SCD knowledge was significantly associated with the parent's age (*p* = 0.005), age of the children (*p* < 0.001) and education level (parents with primary, *p* = 0.014; secondary, *p* = 0.052; tertiary education, *p* = 0.065, had higher SCD knowledge scores compared to those with no formal education) in the univariable analyses. SCD knowledge was not associated with health literacy (*p* = 0.359). In the multivariable analyses, only the child's age (*p* = 0.001) and education level remained significantly associated (primary, *p* = 0.010; secondary, *p* = 0.061; tertiary, *p* = 0.036) with the SCD knowledge score. Table 2 presents the findings from the univariable and multivariable analyses.

**TABLE 2:** Association between sickle cell disease (SCD) knowledge and socio‐demographic characteristics.

	Univariate		Multivariable	
	Coefficient (SD)	*p*‐Value	Coefficient (SD)	*p*‐Value
Parent's age	**0.087**	**0.005** ^*^	−0.004	0.917
Child's age	**0.256**	**0.000** ^**^	**0.266**	**0.001** ^**^
Parent's gender (female)	−0.222	0.751		
Child's gender (female)	−0.056	0.881		
Child's genotype (HbSS)	−0.338	0.437		
Parent's education level				
Primary	**2.198**	0.014^*^	**2.138**	**0.010** ^*^
Secondary	1.667	0.052	1.489	0.061
Tertiary	1.755	0.065	**1.856**	**0.036** ^*^
Parent's employment level				
Self‐employment	−0.400	0.383		
Unemployed	0.721	0.200		
Parent's health literacy score (NVS)	0.359	0.054		

*Note*: No education—reference category for education. HbSC—reference category for child's genotype. Salaried employment—reference category for employment. Male—reference category for parents's and child's gender.

Abbreviations: NVS, newest vital sign; SD, standard deviation.

^*^ *p* ≤ 0.05.

^**^ *p* ≤ 0.001.

### 3.3 Association with hospital admissions and painful episodes

We ran univariable analyses to analyse associations of hospital admissions and frequency of occurrence of painful episodes with SCD knowledge, health literacy scores and socio‐demographic factors and genotype (table 3). Hospital admissions were significantly associated with SCD knowledge (*p* = 0.002), child's age (*p* = 0.003) and employment status (children of participants who were self‐employed recorded more hospital admissions (*p* = 0.044) than those with salaried employment). Health literacy scores, gender, education level and the child's genotype were not associated with hospital admissions. In the multivariable analyses, hospital admissions remained significantly associated with SCD knowledge (*p* = 0.034) and child's age (*p* = 0.039). However, employment status was no longer significant (self‐employed, *p* = 0.067). The child's age was identified as a confounder in the associations between SCD knowledge and hospital admissions. In the univariable analysis between the frequency of occurrence of painful episodes and SCD Knowledge, health literacy scores and  socio‐demographicfactors, only parental age was found to be positievely associated (*p* = 0.041; Table 3).

**TABLE 3:** Association between hospital admissions, sickle cell disease (SCD) knowledge, health literacy levels and demographic factors (Poisson regression).

	Hospital admissions				Painful episodes	
	Univariable		Multivariable		Univariable	
	Coefficient	*p*‐Value	Coefficient	*p*‐Value	Coefficient	*p*‐Value
SCD knowledge	**−0.145**	**0.002** ^*^	**−0.095**	0.034*	−0.026	0.315
NVS	0.007	0.940			0.059	0.224
Parent's age	−0.027	0.108			0.014	0.041*
Child's age	**−0.096**	**0.003** ^*^	**−0.051**	**0.039** ^*^	0.011	0.439
Parent's gender (female)	0.054	0.835			−0.136	0.282
Child's gender (female)	−0.095	0.638			0.033	0.715
Parent's education						
Primary	−0.442	0.388			−0.065	0.731
Secondary	−0.618	0.208			−0.191	0.295
Tertiary	−0.502	0.339			−0.239	0.233
Parent's employment						
Self‐employment	**0.594**	**0.044** ^*^	0.337	0.067	−0.059	0.615
Unemployed	−0.234	0.538	0.275	0.218	0.100	0.459
Child's genotype (HbSS)	−0.177	0.494			0.035	0.733

*Note*: No education—reference category for education. Salaried employment—reference category for employment. Male—reference category for parents's and child's gender. HbSC—reference category for child's genotype.

Abbreviations: NVS, newest vital sign; SCD, sickle cell disease.

^*^ *p* ≤ 0.05.

^**^ *p* ≤ 0.001.

## 4 DISCUSSION

The present study described SCD knowledge and health literacy among parents of children with SCD in Benin. Additionally, we investigated their association with hospitalisations and frequency of occurrence of painful episodes while controlling for socio‐demographic factors and genotype.

We found that SCD knowledge and health literacy were rather limited in many parents. This finding is supported by studies in various countries. A study among parents of children with SCD conducted in Ghana, West Africa, showed knowledge gaps in signs, symptoms and management of the condition [22]. One study carried out in the Netherlands also found a high prevalence of low health literacy in parents of children with SCD [23]. Most of the participating parents were non‐western migrants. In contrast, in a US study among parents attending an urban paediatric SCD clinic, limited health literacy was not as prevalent [24]. Inadequate health literacy is particularly common among populations with migrant backgrounds [25]. These contrasting results suggest that the prevalence of limited health literacy varies across different populations and healthcare settings.

Our findings showed that parents’ SCD knowledge was associated with the age of their children and their own educational level. Parents of older children with SCD were more knowledgeable than parents of younger children. This can be explained by the experiential learning that the parents acquire over time, and by the repeated follow‐up care visits where they get more information and education about the child's condition. Compared to parents with no formal education, parents with higher education levels had higher disease knowledge except for secondary level, which did not reach statistical significance. This is in accordance with the literature where individuals with higher levels of education are more likely to have better knowledge of their disease [9].

Surprisingly, health literacy was not found to be significantly associated with SCD knowledge. This is in contrast to previous results from other studies, which showed that health literacy was associated with the knowledge about chronic diseases [14, 24, 26]. Health literacy is necessary to receive information and education about a chronic disease [24]. Therefore, it is expected that individuals with a high health literacy level will have a better disease knowledge level. In our sample, the only factor that remained significantly associated with the SCD knowledge was the age of the children and parents’ education level. This might indicate that parents have specific disease knowledge on their child's disease, probably because of continued education by doctors and by their own experience of caring for their child. Continued education provided during follow‐up thus seems of great importance.

Hospital admissions were not found to be associated with health literacy. However, parents with lower SCD knowledge, younger children and those who were self‐employed reported more hospital admissions. With the exception of employment status, these associations remained significant. These results are similar to those of a US study in which disease knowledge but not health literacy, was associated with visits to the emergency department [9].This finding adds to the conflicting literature on the association between health literacy and health outcomes in SCD. Although inadequate health literacy is believed to increase the risk of hospital admissions in general [27, 28, 29], other studies have also shown that health literacy and hospital admission were not correlated [9, 30].

Our findings suggest that disease‐specific knowledge in caregivers of a child with a chronic condition such as SCD can be useful in preventing repeated hospital admissions. This may be connected to early recognition of symptoms and taking the necessary measures to prevent complications that necessitate hospitalisation and overall management in general [9]. It could also be related to medication adherence [9], although this was not assessed in this study. Parents of children with SCD can benefit from dedicated education programs to help improve the knowledge of their children's condition [31].

We also observed that the level of educational attainment and employment status were not associated with hospital admissions. Both education attainment and employment status are common indicators of socio‐economic status [32], which is generally believed to have positive impacts on health outcomes in general. More specifically, employment status has also been shown to be associated with hospital admissions in patients with SCD [33]. These results are instructive for the specific context in Benin. Regardless of education levels, parents of children with chronic conditions such as SCD can be equipped with adequate knowledge of the disease in either lay or local languages.

No significant associations were found between disease knowledge and the frequency of occurrence of painful episodes. This was unexpected considering the observed association between SCD knowledge and hospitalisations. It is possible that the frequency of occurrence of painful episodes was underestimated by the parents as sometimes parents may be unaware that their children experienced painful episodes [22].

An important strength of this study is that the questionnaire used to assess SCD knowledge was developed by a multidisciplinary team involving health researchers, general practitioners and SCD specialists in Benin. This ensures that relevant contexts in Benin are accounted for, which are absent with other SCD knowledge questionnaires that had been used previously. A key limitation to this study is the sample bias arising from the use of convenience sample. It is possible that those presenting at the clinic for routine consultations are those whose children experience more severe forms or symptoms of SCD. However, an alternative interpretation suggests that parents with a higher level of awareness regarding the health of their children may have been more likely to attend regular clinic visits. Their proactive approach to seeking routine medical care could reflect a greater understanding of the importance of managing their children's health conditions. It is important to consider both perspectives when interpreting the findings. Despite the sample bias limitation, it is noteworthy that most participants in our study recorded few hospital admissions, which may indicate a relatively well‐managed health condition among the included participants. Another limitation to the study is that both instruments have not been specifically validated in the population in Benin. This suggests that future studies can modify the questionnaire and evaluate the psychometric properties. Future research should consider other clinical characteristics associated with SCD crisis and hospital admissions.

## 5 CONCLUSION

This study provides fundamental knowledge for understanding the current situation of SCD in a country such as Benin in West Africa. Targeted education to improve disease‐related knowledge for parents of children with chronic diseases such as SCD is very important. Continuous disease‐specific education will help them better identify and prevent the occurrence of symptoms and improve overall disease management.

## AUTHOR CONTRIBUTIONS

Study design was made by Bonaventure G. Ikediashi and Gisela Michel. Data analysis was carried out by Bonaventure G. Ikediashi and Cristina Ehrmann. Manuscript was written by Bonaventure G. Ikediashi and revised by Cristina Ehrmann, Selma Gomez and Gisela Michel.

## CONFLICT OF INTEREST STATEMENT

The authors declare they have no conflicts of interest.

## ETHICS STATEMENT

This cross‐sectional study among parents of children with SCD was approved by the National Ethics Committee in Health Research in Benin (CNERS Avis No. 43).

## PATIENT CONSENT STATEMENT

Patients provided written informed consent before data were collected.

## PERMISSION TO REPRODUCE MATERIAL FROM OTHER SOURCES

N/A.

## CLINICAL TRIAL REGISTRATION (INCLUDING TRIAL NUMBER)

N/A.

## Supporting information

Supporting Information: Click here for additional data file.

## Data Availability

The data that supports the findings of this study are available in the supplementary material of this article.
